# Editorial: Case reports in urothelial cancer

**DOI:** 10.3389/fonc.2023.1150420

**Published:** 2023-02-07

**Authors:** Mario W. Kramer

**Affiliations:** Department of Urology, University Hospital Schleswig-Holstein, Luebeck, Germany

**Keywords:** case report, urothelial cancer, neuroendocrine tumor, checkpoint inhibition, robotic surgery

Urothelial cancer is a difficult type of tumour entity. It may be harmless and easily treated, it can be bothersome with constantly recurrent disease affecting quality of life and it is lethal once progressed to metastatic disease. The course of this disease may be assumed but often is not anticipated. Treatment algorithms vary from office-based tumour fulguration over cystectomy and or nephroureterectomy to complex systemic therapy. During the past years new treatment options have been developed such immunotherapy or drug conjugates hereby increasing life expectancy in a still deadly disease.

Although guidelines have been blown up with information, still there is lack of knowledge in many cases. Examples? We used to treat urothelial cancer with predominant neuroendocrine component with primary chemotherapy but most patients only survived months after cystectomy. Changing to early cystectomy in low volume disease has provided better results. A young woman with pulmonary metastases has not responded to chemotherapy or immunotherapy. After several surgical treatments she achieved long term recurrence free disease. A patient had massive urothelial tumours in both upper tracts and in his bladder but never developed metastases. Every Urologist and Uro-Oncologist has similar stories to tell. These are patients we learn from each other but you will never find answers within guidelines as they rely on randomized controlled trials or at least on big cohort studies.

The reader of this issue may find some answers that have not been told. Therefore, I encourage everyone to read through the following articles:

Sharma et al. present the rare case of a 19-year-old male with advanced urothelial carcinoma of the bladder. They performed whole-exome sequencing to identify potential treatment targets hereby presenting potential driver mutations and raising the question whether mutations in genes involved in ion channels may be engraving tumours.

Another highly interesting case is reported by Xu et al. A patient with neuroendocrine carcinoma of the ureter colliding with squamous cell carcinoma is discussed respecting current literature of only 16 previous cases worldwide. In this case the combination of surgery and systemic therapy (cisplatin/etoposide) has provided prolonged survival. Another small cell neuroendocrine carcinoma case is presented by Qing et al. They describe the rapid disease progression despite surgery and adjuvant systematic therapy including PD-L1 immuno-checkpoint inhibition (ICI) combined with radiotherapy.

Systemic treatment of urothelial cancer consists of platinum-based chemotherapy and ICI therapy. Up to date, the antibody-drug conjugate Enfortumab vedotin has been approved by FDA and EMA for treatment after failure of the two previously mentioned therapies. Especially the use of immuno-checkpoint inhibitors may lead to long-term survival in metastatic urothelial carcinoma which has rarely been described before. New checkpoint inhibitors have been developed and are now increasingly used. Therefore, we present two case reports in which treatment success by using new PD-1 inhibitors are reported. Li et al. administered the PD-1 inhibitor Tislelizumab that showed remission in an isolated renal calyceal urothelial carcinoma. Zan et al. used the PD-1 inhibitor Toripalimab combined with the multi-targeting tyrosine kinase inhibitor Anlotinib in last line sequence which resulted in long-term clinical response for over 25 months in a patient with metastatic disease.

Robot assisted surgery is increasingly used in complex situations. Cai et al. present a case of a 56-year old male patient who developed recurrent disease in the ureteral cutaneous stoma after radical cystectomy. Interestingly they performed a completely intracorporal resection of the tumour and ileal conduit surgery. They describe their procedure step by step.

I hope you find this selection of case reports worth reading.

## Author contributions

The author confirms being the sole contributor of this work and has approved it for publication.

